# Light discomfort thresholds in patients with senile cataract versus pseudophakic subjects

**DOI:** 10.1007/s00417-025-06941-3

**Published:** 2025-08-12

**Authors:** Filippo Lixi, Claudia Corda, Giulia Coco, Giovanna Carnovale-Scalzo, Alessandra Mancini, Maria Angela Romeo, Vincenzo Scorcia, Giuseppe Giannaccare

**Affiliations:** 1 Department of Surgical Sciences, Eye Clinic, University of Cagliari, Cagliari, Italy; 2 Ophthalmology, Department of Clinical Sciences and Translational Medicine, University of Rome Tor Vergata, Rome, Italy; 3 Department of Ophthalmology, University Magna Graecia of Catanzaro, Catanzaro, Italy

**Keywords:** Light sensitivity, Photophobia, Glare, Light discomfort thresholds, Cataract surgery

## Abstract

**Purpose:**

To compare light discomfort thresholds in patients with senile cataract versus pseudophakic subjects.

**Methods:**

In this cross-sectional, multicenter, controlled study light discomfort thresholds were measured in both eyes of patients with senile cataract and in sex- and age-matched pseudophakic subjects using the Lumiz 100 device (Essilor International, Paris, France) under three different lighting conditions: continuous warm, continuous cold and flashing warm. For continuous increase, light starts at 25 lx for 5 s and increases every second by 20%; for discontinuous increases, light starts at 10 lx for 5 s followed by increases to 25 lx for half a second and then decreases back to 10 lx for 2 s, before a 44% increase from the previous flash using warm light. Age, gender, pupil diameter, self-reported light sensitivity, iris color, eyeglass use, visual acuity, lens status and cataract grade were analyzed and correlated with light discomfort thresholds.

**Results:**

A total of 48 participants (28 men, 20 women; mean age 72.92 ± 9.05 years) were included. Patients with senile cataract exhibited statistically significant lower discomfort thresholds for all measurements compared to pseudophakic subjects (respectively, 3.39 ± 0.66 log10[lux] vs. 3.81 ± 0.46 [*p* = 0.014] for continuous warm; 3.44 ± 0.64 vs. 3.82 ± 0.42 [*p* = 0.025] for continuous cold; 3.14 ± 0.59 vs. 3.81 ± 0.31 [*p* < 0.001] for flashing warm). Apart for lens status, no other variables influenced light discomfort thresholds.

**Conclusion:**

The Lumiz 100 enabled the evaluation of light discomfort thresholds in patients with different lens status, highlighting a higher light sensitivity in patients with senile cataract compared to pseudophakic subjects under all lighting conditions.

## Key messages

*****What is known***:**

- Glare and light sensitivity represent symptoms commonly reported by patients affected by senile cataract.
- Previous studies analyzing light sensitivity largely relied on subjective measures such as questionnaires and scales.

*****What is new***:**

- This study shows that Lumiz 100 device can be used as a tool for measuring light sensitivity thresholds in patients with different lens status.
- Patients with senile cataract presented a significant higher light sensitivity compared to matched pseudophakic subjects. Light sensitivity thresholds could be a further parameter to consider when evaluating the need and the timing for cataract surgery.

## Introduction

Light sensitivity, also called photophobia, and glare represent sensory conditions associated with various underlying disorders that may present an altered neuronal pathway [1, 2]. Although the term photophobia lacks a precise definition, it can be described as an increased sensitivity to normal levels of illumination [3]. Glare is a phenomenon caused by light entering the eye that does not contribute to vision; it is generally considered as a combination of disability glare and discomfort glare, which are usually concurrent but can also present independently [3]. Specifically, disability glare is described as the reduction of retinal image contrast due to light scatter within the eye [4], while discomfort glare refers to visual annoyance caused by bright light sources [3].

The crystalline lens plays a crucial role in focusing light onto the retina, enabling the eye to create sharp and detailed images for the brain [5]. Age-related biochemical and structural alterations in the lens, can compromise its transparency and contribute to the development of senile cataract. Beyond to reduced visual acuity and contrast sensitivity [6], progressive optical changes related to cataract formation can determine impaired light spatial properties and wavelength distribution of the retinal image [7], monocular double vision [8], myopic shift [9], glare, and increased light sensitivity [6, 7].

Although different strategies including questionaries [10], scales [11, 12] and tools [13–15] have been employed over the last years to evaluate light sensitivity, the wide discrepancy among them and the lack of quantitative measurements represent a current gap. Recently, a novel device (Lumiz 100, Essilor International, Paris, France) able to offer rapid, reliable, and safe evaluation of light discomfort thresholds has been introduced on the market. Preliminary studies on healthy individuals have demonstrated the feasibility of its use as a clinical tool for assessing light sensitivity [16, 17].

The present study aimed at comparing light discomfort thresholds between patients with senile cataract and pseudophakic subjects in different lighting conditions. Highlighting different thresholds between the two groups may enhance the ability to quantify visual disturbances other than decreased visual acuity in patients with senile cataract.

## Methods

In this cross-sectional, controlled study, participants were consecutively recruited at two University Eye Clinics. Inclusion criteria were individuals aged 60 years or older with bilateral symmetrical senile cataract defined by a minimum Lens Opacities Classification System (LOCS) III score of 2 [18], or with bilateral pseudophakia after a recent (3–12 months) uneventful phacoemulsification with implantation of monofocal, single-piece, clear aspheric acrylic intraocular lens (IOL) (AcrySof SA60AT; Alcon Laboratories, Inc., Fort Worth, TX).

Exclusion criteria included a history of relevant ocular diseases, uncontrolled systemic or ocular conditions, refractive error (hyperopia, myopia, astigmatism) > 1.5 diopters, irregular corneal shape, posterior capsular opacification (only for pseudophakic subjects), migraine, epilepsy and use of medications with a known link with light sensitivity (e.g. non-steroidal anti-inflammatory drugs, anticonvulsants, anti-rheumatic drugs, chemotherapy, antibiotics, antiarrhythmic, atropine, antipsychotics, anti-ulcer drugs, immunosuppressants). Ethical approval for the study was obtained from the Ethics Committee “Comitato Etico Territoriale Lazio Area 2” (approval date 13-02-2025). Informed consent was obtained from all participants, and the study was conducted in accordance with the tenets of the Declaration of Helsinki.

### Data acquisition

After having screened for eligibility, participants fulfilling study criteria underwent a comprehensive ophthalmologic examination for the collection of the following data: age, sex, history sunglasses use, self-reported light sensitivity, best-corrected visual acuity (BCVA), slit-lamp exam for cataract grading and iris color analysis (dark vs. light), pupil diameter, and light discomfort thresholds measured by means of Lumiz 100 device (Essilor International, Paris, France).

Cataract was classified using the LOCS III under pupil dilation with tropicamide 0.5% and phenylephrine hydrochloride 10% eye drops (Visumidriatic fenilefrina, Visufarma S.p.a., Italy) by two trained physicians (F.L. and C.C.) for the evaluation of nuclear color/opalescence (0.1–6.9), cortical cataract (0.1–5.9), and posterior subcapsular cataract (0.1–5.9) [18]. For each patient, the overall cataract score as the mean of both eyes’ cataract grading was calculated for statistical analysis. In pseudophakic subjects, a complete ophthalmic examination was carried out to exclude the presence of posterior capsule opacification or any other complication related to the previous lens surgery.

The OA-2000 (Tomey GmbH, Nagoya, Japan) was used to measure pupil size under dim lighting conditions. The mean pupil diameter of both eyes was calculated for each patient and included in the statistical analysis.

Visual acuity, assessed using Snellen charts, was converted to logMAR for analysis. The mean visual acuity, considered as the average of the visual acuities of both eyes, was calculated.

Participants completed a validated questionnaire to rate their self-perception of light sensitivity by responding to the question: “Do you feel sensitive to light?” with one of the following options: (1) Yes, a lot; (2) Yes, a bit; (3) No, not really; (4) No, not at all [17].

Light discomfort thresholds were measured using the Lumiz 100, a portable device that provides uniform diffuse illumination across the user’s visual field. Since photosensitivity has a logarithmic relationship with light intensity, the lux thresholds were log-transformed, and all statistical analyses were conducted using the log10 (lux) thresholds. A tablet application controls light intensity ranging from 10 lx (log10[lux] = 1) to 10,211 lx (log10[lux] = 4.01) at eye level. If a threshold was not reached at 10,211 lx, the subsequent level (12,253 lx) was arbitrarily assigned. Thresholds were determined for two levels of discomfort under three lighting conditions simulating different everyday environments: two with continuous light increases and one with discontinuous increases. For continuous increase, light starts at 25 lx for 5 s and increases every second using a 20% increase step, using either warm light (4000 K color temperature) mimicking natural light or cold light (6500 K color temperature) mimicking artificial light. For discontinuous increases, light starts at 10 lx for 5 s followed by increases to 25 lx for half a second and then decreases back to 10 lx for 2 s, before a 44% increase from the previous flash using warm light (4000 K). This flashing increase does not provide enough time for the visual system to adapt and has been chosen to reflect the most bothering situations in everyday life. Participants were instructed to press a button twice to signal discomfort levels. The first level, called the “just perceptible” discomfort threshold, occurs when the discomfort is initially noticed, and participants feel mild symptoms such as “tension in the eyelids or tingling.” The second level, the “really disturbing” discomfort threshold, is reported when the discomfort becomes bothersome requiring effort to keep their eyes open. The mean of these two levels represented the mean threshold for each of the three lighting conditions. Total light sensitivity threshold was calculated as the mean of six logarithmic illumination thresholds. The protocol and measurement reliability have been detailed in previous studies [16, 17].

### Statistical analysis

Statistical analysis was conducted using SPSS for Macintosh software (version 30.0.0.0, SPSS, Inc.) Means ± standard deviations (SDs) were calculated for numerical continuous variables, while percent distributions were presented for categorical data. The distribution of variables was evaluated using the Shapiro-Wilk test. Fisher’s exact test was employed to compare categorical variables. Parametric (t-test) and non-parametric (Mann–Whitney U test and Kruskal–Wallis test) tests were used to compare normally and non-normally distributed variables among groups, respectively. The relationships between the total light sensitivity and other numerical data were evaluated using Spearman’s correlation test. A post hoc power analysis was conducted by using G*Power software (version 3.1.9.6) to assess the statistical power of the study. Based on the difference in the primary outcome (total light sensitivity threshold 3.32 ± 0.59 log10[lux] and 3.81 ± 0.36 log10[lux]) respectively between the two groups (*n* = 24), the effect size (Cohen’s d) was calculated as approximately 1.0. Using a two-sided test with an alpha level of 0.05, the calculated power was approximately 0.92 (92%), indicating a high likelihood of detecting a true difference between the groups. This indicates that our sample size was adequate to detect the observed difference. A p-value of less than 0.05 was considered statistically significant.

## Results

A total of 96 eyes of 48 patients (28 men, 20 women; mean age 72.92 ± 9.05 years) were included in the analysis. All patients were Caucasians; 41 (85.4%) patients had dark irises while 7 (14.6%) had light irises. Twenty (41.7%) participants were sunglasses users while 28 (68.3%) reported to not use sunglasses. The mean pupil diameter was 4.82 ± 0.52 mm. Eleven (22.9%) patients rated their self-perception of light sensitivity as 1, 13 (27.1%) as 2, 17 (35.4%) as 3 and 7 (14.6%) as 4. Mean BCVA was 0.23 ± 0.24 logMAR. Mean continuous warm sensitivity threshold was 3.60 ± 0.60 log10[lux]. Mean continuous cold sensitivity threshold was 3.63 ± 0.57 log10[lux]. Mean flashing warm sensitivity threshold was 3.47 ± 0.57 log10[lux]. Total light sensitivity threshold was 3.57 ± 0.55 log10[lux]. Differences in the total light sensitivity threshold across the categorical variables were analyzed in Table 1.

**Table 1:** Total light sensitivity thresholds according to categorical variables

	All patients *n* (%)	Mean total light sensitivity threshold (SD)	*p*-value
Gender			0.571^‡^
Male	28 (58.3%)	3.61 (0.53)	
Female	20 (41.7%)	3.51 (0.57)	
Iris color			0.689^‡^
Dark	41 (85.4%)	3.54 (0.57)	
Light	28 (14.6%)	3.71 (0.34)	
Sunglasses user			0.550^‡^
Yes	20 (41.7%)	3.63 (0.48)	
No	28 (58.3%)	3.52 (0.59)	
Self-perception of light sensitivity			0.337^#^
Yes, a lot	11 (22.9%)	3.37 (0.65)	
Yes, a bit	13 (27.1%)	3.52 (0.50)	
No, not really	17 (35.4%)	3.72 (0.44)	
No, not at all	7 (14.6%)	3.60 (0.69)	
Lens status			< 0.001^‡*^
Pseudophakic	24 (50.0%)	3.81 (0.36)	
Cataract	24 (50.0%)	3.32 (0.59)	
Cataract type			0.605^#^
Nuclear Cataract	13 (27.1%)	3.24 (0.58)	
Cortical Cataract	5 (10.4%)	3.33 (0.70)	
Posterior Subcapsular Cataract	6 (12.5%)	3.48 (0.61)	

‡ = Mann–Whitney U test; # = Kruskal-Wallis test.

There was no effect of gender, iris color, sunglasses use and self-perception of light sensitivity on the total light sensitivity thresholds. On the other hand, the lens status significantly influenced the total light sensitivity threshold. Mean total light sensitivity threshold was significantly lower in patients with senile cataract compared to pseudophakic subjects (3.32 ± 0.59 log10[lux] vs. 3.81 ± 0.36 log10[lux], p = < 0.001, Mann–Whitney U test).

Furthermore, the total light sensitivity threshold and the continuous variables investigated were tested for a possible correlation. No significant results were observed for age (*R* = 0.278, *p* = 0.056, Spearman’s test), BCVA (*R*=−0.022, *p* = 0.884) and pupil diameter (*R*=−0.177, *p* = 0.228).

Patients with senile cataract (cataract group, *n* = 24) were compared with sex- and age-matched subjects who already underwent phacoemulsification with monofocal IOL implantation (pseudophakic group, *n* = 24). Among patients with cataract, 13 (27.1%) had nuclear cataracts, 5 (10.4%) had cortical cataracts and 6 (12.5%) had posterior subcapsular cataracts. The overall cataract score was 3.72 ± 1.00. The characteristics of the two groups are displayed in Table 2.

**Table 2:** Demographic and clinical characteristics of cataract and pseudophakic groups

	Cataract group (*n* = 24) *n* (%)	Pseudophakic group (*n* = 24) *n* (%)	*p*-value
Gender			0.142^*φ*^
Male	11 (45.8%)	17 (70.8%)	
Female	13 (54.2%)	7 (29.2%)	
Mean age (SD)	71.00 (8.31)	74.83 (9.52)	0.144^†^
Mean BCVA (SD)	0.33 (0.24)	0.14 (0.14)	0.002^‡*^
Iris color			1^*φ*^
Dark	21 (87.5%)	20 (83.3%)	
Light	3 (12.5%)	4 (16.7%)	
Owner of sunglasses			1^*φ*^
Yes	10 (41.7%)	10 (41.7%)	
No	14 (58.3%)	14 (58.3%)	
Mean pupil diameter (SD)	4.90 (0.52)	4.73 (0.51)	0.364^‡^
Self-perception of light sensitivity			0.089^χ^
Yes, a lot	8 (33.3%)	3 (12.5%)	
Yes, a bit	6 (25.0%)	7 (29.2%)	
No, not really	5 (20.8%)	12 (50.0%)	
No, not at all	5 (20.8%)	2 (8.3%)	
Mean continuous warm sensitivity threshold	3.39 (0.66)	3.81 (0.46)	0.014^‡*^
Mean continuous cold sensitivity threshold	3.44 (0.64)	3.82 (0.42)	0.025^‡*^
Mean flashing warm sensitivity threshold	3.14 (0.59)	3.81 (0.31)	< 0.001^‡*^

φ = Fisher’s exact test; † = t test; ‡ = Mann–Whitney U test; χ = Chi-squared test. BCVA = Best-corrected visual acuity.

**Fig. 1:**
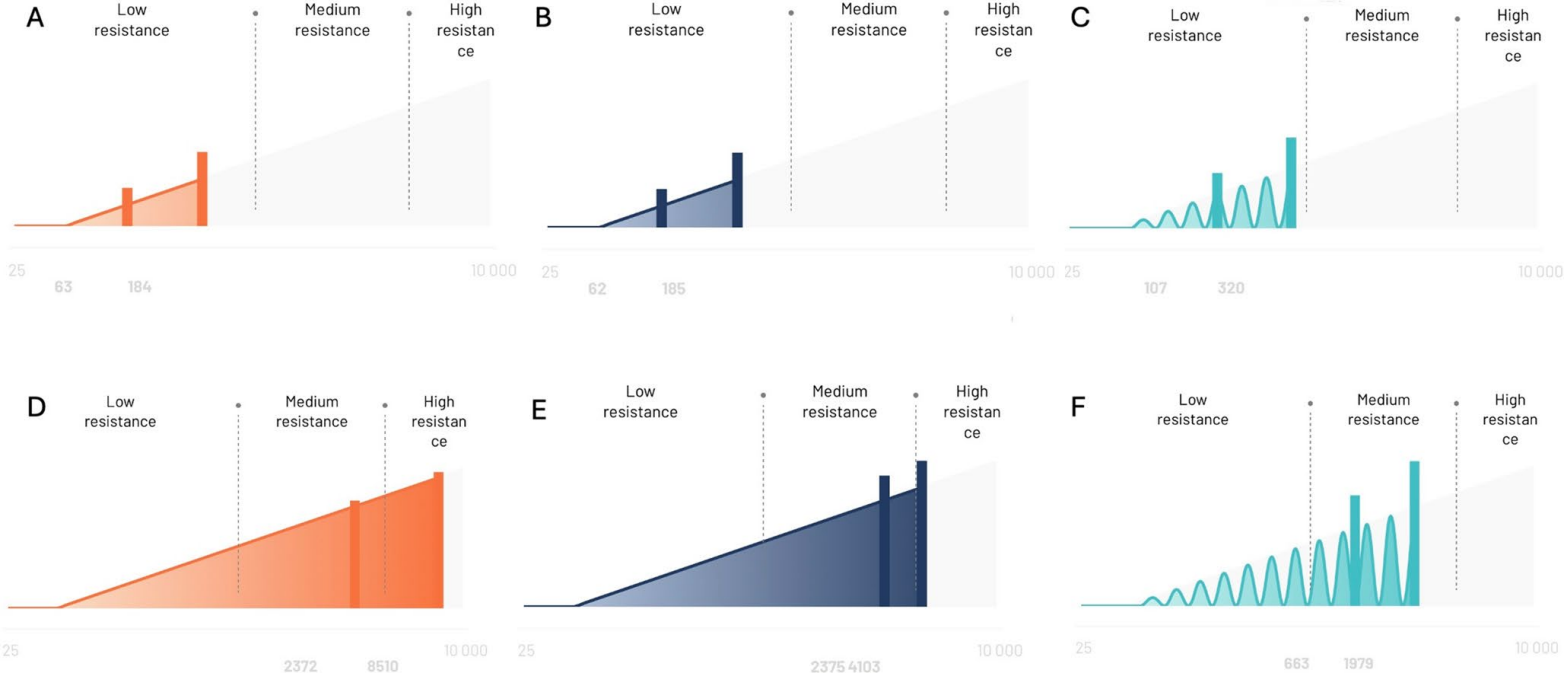
Light discomfort thresholds for continuous warm, continuous cold, and flashing warm in a representative patient with senile cataract (nuclear cataract, NC/NO grade 4.6 according to LOCS III) (respectively, **A**-**C**) and in a representative patient with pseudophakia (respectively, **D**-**F**). For each lighting condition, the first vertical line indicates the onset of a just perceptible discomfort, while the second one represents the onset of a really disturbing discomfort

Gender, mean age, iris color, sunglasses use, pupil diameter and self-perception of light sensitivity showed no significant differences between the two groups. Patients with cataract exhibited a significantly worse BCVA than pseudophakic individuals (0.33 ± 0.24 logMAR vs. 0.14 ± 0.14 logMAR, *p* = 0.002). Cataract group showed significantly lower discomfort thresholds compared to matched pseudophakic subjects for all light condition measurements (respectively, 3.39 ± 0.66 log10[lux] vs. 3.81 ± 0.46 log10[lux], *p* = 0.014 for continuous warm; 3.44 ± 0.64 log10[lux] vs. 3.82 ± 0.42 log10[lux], *p* = 0.025 for continuous cold; 3.14 ± 0.59 log10[lux] vs. 3.81 ± 0.31 log10[lux], *p* < 0.001 for flashing warm). Figure 1 shows the different resistance to the light in a representative patient with senile cataract (top) and in a representative pseudophakic subject (bottom).

No significant differences were demonstrated in total light sensitivity threshold across each categorical data analyzed in the two groups.

For each group, the total light sensitivity threshold and the continuous variables were tested for a possible correlation (Table 3).

**Table 3:** Correlation between total light sensitivity threshold in each group with numerical continuous variables

	Total light sensitivity threshold	Mean age	Mean BCVA	Mean pupil diameter	Mean Cataract Grading
Cataract group	Patients N	24	24	24	24
	Spearman’s R	24	−0.114	−0.134	−0.252
	*p*-value	0.668	0.597	0.532	0.235
Pseudophakic group	Patients N	24	24	24	/
	Spearman’s R	0.306	0.540	−0.158	/
	*p*-value	0.146	0.006^*^	0.462	/

*BCVA *Best-corrected visual acuity

For the cataract group, no significant correlation was observed among the total light sensitivity thresholds and the other continuous variables; conversely, in the pseudophakic group, a significant positive correlation was found between the total light sensitivity thresholds and the mean BCVA (*r* = 0.540, *p* = 0.006).

## Discussion

The present study highlighted the utility of the Lumiz 100 in assessing light discomfort thresholds in patients with different lens status, revealing significant differences in light sensitivity between patients with senile cataract and pseudophakic subjects.

Light sensitivity and glare are not well-defined conditions whose precise quantification and adequate treatment remain challenging [19, 20]. Several ocular and neurological conditions have been associated with heightened light sensitivity [1, 6, 17, 19]. Therefore, evaluating thresholds and their changes according to different variables could greatly benefit clinical practice. Recent studies showed that the Lumiz 100 device enables the assessment of light sensitivity thresholds through a quick, safe, and straightforward procedure, providing insights into how these thresholds may vary in relation to different physiological factors in healthy patients [16, 17].

In our study, gender did not influence total light sensitivity thresholds. While Marié et al. [17] observed lower discomfort thresholds in females in a larger sample using the same device, several other studies reported no significant differences between males and females [13, 15, 21, 22]. Although these conflicting results underscore the importance of further investigations, this is not surprising since it is generally accepted that gender does not significantly affect the perception of light [22].

Iris color did not appear to influence total light sensitivity thresholds, in agreement with prior studies that showed minimal or no effect of iris pigmentation on light discomfort [17, 21, 22]. Similarly, we observed no differences in light sensitivity between individuals who regularly use sunglasses and those who do not, contrasting with findings by Marié and colleagues [17]. Variations in sunglass types and criteria for identifying users may account for these discrepancies.

Discomfort thresholds and patients’ self-reported light sensitivity did not align in our analysis. While some studies have linked self-assessed light sensitivity to lower discomfort thresholds [18, 20], others reported no significant association [22, 23]. These inconsistencies highlight the subjective and qualitative nature of questionnaire and suggest the importance of using tools for the quantitative measurements of light sensitivity, such as the Lumiz 100, able to acquire more reliable and standardized data about patient’ light complaints.

A significant difference in total light sensitivity thresholds was found according to lens status. Cataract remains the leading cause of visual impairment worldwide [5]. Lens opacities can alter light transmittance, increasing forward light scattering to the retina, and causing disturbing glare effects up to photophobia which ultimately impair daily activities and social functioning [6, 24]. Given the challenges in quantifying these subjective conditions, indication for cataract surgery focuses heavily on lens status evaluation at slit-lamp examination and reduced vision at visual acuity test [24]. In addition to visual recovery and improvement of overall cognitive function [25], cataract extraction has been reported to reduce glare and light sensitivity [6, 7, 26]. Indeed, by removing an opaque and imperfect lens and implanting a clear IOL, the intraocular light scatter can be mitigated [2, 6]. However, since the IOL has a higher light transmittance than the natural lens, it can potentially contribute to increase glare under certain conditions [27, 28]. It is important to point out that other factors, such as variations in IOL materials and older surgical techniques, may also have influenced glare levels in previous studies [27, 28]. Consistently with literature [6, 7, 26], pseudophakic subjects showed a significant higher sensitivity thresholds for each light condition compared to patients with senile cataract. Furthermore, as previously reported [29], the type of cataract did not have a significant impact on glare discomfort in our analysis.

Apart from lens status, none of the physiological variables explored appeared to influence light sensitivity thresholds. These findings suggest that light scattering through lens opacities plays a dominant role in glare and photophobia, which are otherwise more closely related to impaired neuronal inputs and cerebral processing mechanisms rather than physiological factors [17, 30]. Notably, more sensitive individuals appeared to have greater neuronal activity in the visual cortex, regardless of light intensity levels [30]. As a matter of fact, several neurological conditions including migraines, blepharospasm, and traumatic brain injuries appeared to be related to photophobia [1].

Controversial data are available about the impact of age on light sensitivity. Although there was a trend toward a positive correlation between age and light sensitivity thresholds, this relationship was not statistically significant in our cohort. This finding aligns with prior research suggesting that age does not significantly influence light sensitivity thresholds [6, 17, 22]. However, our cohort, interestingly, demonstrated a higher mean total light sensitivity threshold compared to the values reported by Marié et al. [17]. Differences in age distribution may explain this disparity, as younger individuals are described to be more sensitive to light than older people [31, 32]. It can be supposed that delayed responses to the Lumiz 100 examination and consequently higher light thresholds observed among elder population can be related to slower proprioceptive and cognitive reactions or to an effort to overcome their physical limits for showing an increased resistance to light.

A possible correlation among the light sensitivity threshold and other parameters such as cataract grading, pupil size and BCVA was investigated. Light scatter increases with cataract progression [33]. Indeed, a negative correlation, despite non statistically significant, was recorded between the light discomfort threshold and cataract grading. Although a possible correlation between pupil size and light discomfort was registered [34], in our study as well as in other reports [17, 22], no correlation was observed between dim light pupil size and discomfort thresholds. The BCVA did not correlate with light sensitivity threshold in patient with cataract; however, a positive correlation in pseudophakic group was recorded. Indeed, in patients which have already removed potentially light scattering media, as visual acuity decreases, the light tolerance increases. Although this finding requires further evaluation, a possible relationship with reduced contrast sensitivity or with diminished neuronal transmission to the cortical visual area can be hypothesized.

This study offers novel insights into the assessment of light sensitivity thresholds in patients with senile cataract and in those who have undergone phacoemulsification with IOL implantation. Nonetheless, some limitations must be acknowledged. Firstly, the cross-sectional design of the study restricts our ability to establish causality between the different factors and light sensitivity thresholds. A longitudinal evaluation in the same patients before and after phacoemulsification would be beneficial to better understand the impact of lens status and surgery. Secondly, the relatively small sample size may have hampered the statistical significance within subgroups (e.g. cataract types). Thirdly, the Lumiz 100 is a device specifically designed to evaluate light sensitivity thresholds binocularly, reflecting real-world visual experience where both eyes are typically stimulated simultaneously. To ensure the reliability and homogeneity of our data, we included only patients with symmetrical ocular status (bilateral similar-graded cataract or bilateral pseudophakia with the same IOL type) and used mean values of both eyes for the study analysis. Patching one eye during examination could have provided unilateral parameters but this use has not been validated and future studies are still required. Lastly, incorporating a more comprehensive and validated symptom questionnaire beyond the used self-reported measure would enhance the correlation between objective measurements and subjective patient experiences.

In conclusion, the present study provides novel and reliable data obtained using the Lumiz 100, offering a significant contribution to current understanding of photosensitivity in both cataract and pseudophakic patients. Our findings suggest that patients with senile cataract have higher light sensitivity compared to pseudophakic subjects, therefore light sensitivity threshold determination could be incorporated in the workflow for evaluating the need and the timing for cataract surgery. Future studies with larger sample size and longitudinal design are desirable to further elucidate the impact of lens status and surgery on light sensitivity. Additionally, expanding this research to different populations, such as patients implanted with multifocal intraocular lenses, may offer valuable insights into the broader applicability of the Lumiz 100 and help integrating light discomfort thresholds into the armamentarium of image quality assessments traditionally based on aberrometry [35].
